# Transcranial Magnetic Stimulation as a Tool to Investigate Motor Cortex Excitability in Sport

**DOI:** 10.3390/brainsci11040432

**Published:** 2021-03-28

**Authors:** Fiorenzo Moscatelli, Antonietta Messina, Anna Valenzano, Vincenzo Monda, Monica Salerno, Francesco Sessa, Ester La Torre, Domenico Tafuri, Alessia Scarinci, Michela Perrella, Gabriella Marsala, Marcellino Monda, Giuseppe Cibelli, Chiara Porro, Giovanni Messina

**Affiliations:** 1 Deparment of Clinical and Experimental Medicine, University of Foggia, 71122 Foggia, Italy; fiorenzo400@gmail.com (F.M.); anna.valenzano@unifg.it (A.V.); francesco.sessa@unifg.it (F.S.); esterlatorre96@gmail.com (E.L.T.); perrellamichela@tiscali.it (M.P.); giuseppe.cibelli@unifg.it (G.C.); chiara.porro@unifg.it (C.P.); 2 Unit of Dietetic and Sport Medicine, Section of Human Physiology, Department of Experimental Medicine, Luigi Vanvitelli University of Campania, 81100 Naples, Italy; antonietta.messina@unicampania.it (A.M.); mondavincenzo@gmail.com (V.M.); marcellino.monda@unicampania.it (M.M.); 3 Department of Medical, Surgical and Advanced Technologies “G.F. Ingrassia”, University of Catania, 95121 Catania, Italy; monica.salerno@unict.it; 4 Department of Motor Sciences and Wellness, University of Naples, 80133 Naples, Italy; domenico.tafuri@uniparthenope.it; 5 Department of Education Sciences, Psychology and Communication, University of Bari, 70121 Bari, Italy; alessia.scarinci@uniba.it; 6 StrutturaComplessa di Farmacia, AziendaOspedaliero-Universitaria, 71122 Foggia, Italy; gabriella.marsala@aspct.it

**Keywords:** corticalexcitability, transcranial magnetic stimulation, motor cortex, TMS

## Abstract

Transcranial magnetic stimulation, since its introduction in 1985, has brought important innovations to the study of cortical excitability as it is a non-invasive method and, therefore, can be used both in healthy and sick subjects. Since the introduction of this cortical stimulation technique, it has been possible to deepen the neurophysiological aspects of motor activation and control. In this narrative review, we want to provide a brief overview regarding TMS as a tool to investigate changes in cortex excitability in athletes and highlight how this tool can be used to investigate the acute and chronic responses of the motor cortex in sport science. The parameters that could be used for the evaluation of cortical excitability and the relative relationship with motor coordination and muscle fatigue, will be also analyzed. Repetitive physical training is generally considered as a principal strategy for acquiring a motor skill, and this process can elicit cortical motor representational changes referred to as use-dependent plasticity. In training settings, physical practice combined with the observation of target movements can enhance cortical excitability and facilitate the process of learning. The data to date suggest that TMS is a valid technique to investigate the changes in motor cortex excitability in trained and untrained subjects. Recently, interest in the possible ergogenic effect of non-invasive brain stimulation in sport is growing and therefore in the future it could be useful to conduct new experiments to evaluate the impact on learning and motor performance of these techniques.

## 1. Introduction 

**Figure 1:**
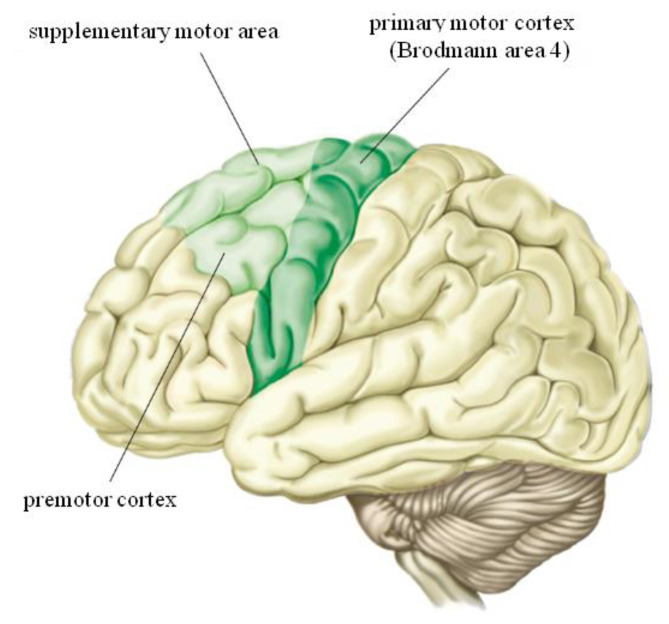
Motor cortex. In this figure shows the position of primary motor cortex (M1), the position of supplementary motor area and the position of premotor cortex. Motor cortex is the region of the cerebral cortex involved in the planning, control, and execution of voluntary movements. Primary motor cortex is the main contributor to generating neural impulses that pass down to the spinal cord and control the execution of movement. Premotor cortex is responsible of motor control.

In adult human in response to various injuries [1], environmental changes [2,3,4,5] and even repetitions of simple movements, the brain show the ability to change its organization through physiological mechanisms, and this phenomenon is called brain plasticity [6,7]. In this contest, brain plasticity enables the nervous system to ensure that proper activation of muscles may be acquired and maintained to serve the behavioral goal [1]. The central nervous system (CNS) is a highly plastic structure that can adapt throughout the lifespan of higher mammals to changes in the environment, however, during the embryonic period, and the first period of life, neural connectivity is under the control of epigenetics. The CNS can adapt throughout the lifespan of mammals and the environmental factors can heavily influence the changes in brain organization, both in the short and long term [8].In the human brain, the cerebral structure that plays a fundamental role in the acquisition and execution of movements is the primary motor cortex (M1). The M1 has numerous connections which respond to external stimuli by adapting and therefore appear to be highly plastic (Figure 1). It would seem that these phenomena of plasticity are due to a neural mechanism through which the different motor skills are encoded by the nervous system. In fact, in support of the above hypothesis, it has been shown that the acquisition of new motor skills modifies the synaptic structure by increasing the number of connections within the motor cortex with the consequent change in motor performance [1]. The M1 is different from other regions of the cerebral cortex in that it is thicker but has a lower cell density. The most important output cells of M1 are the large pyramidal cells in lamina V and the smaller cells in lamina III, and their dendrites show a preferential orientation parallel to the main axis of the precentral gyrus. The M1 contains large corticospinal neurons that send long axons down the spinal cord to synapse onto alpha motor neurons which connect to the target muscle [9]. In the last twenty years, neurostimulation techniques, being non-invasive, have been widely used in order to investigate the human motor cortex and its changes, giving a breakthrough to understand how the brain adapts in response to external stimuli and how these adaptations affectmotor performance.

Repetitive physical training is generally considered as a principal strategy for acquiring a motor skill, and this process can elicit cortical motor representational changes referred to as use-dependent plasticity [3,4,7,10]. It has been shown that in training programs that include physical practice combined with observation of target movements [11], an increase in cortical excitability is observed which can improve and facilitate the learning processes [12]. 

Transcranial Magnetic Stimulation (TMS) permits painless stimulation of the cerebral cortex in humans without requiring open access to the brain, and if used following appropriate guidelines, is devoid of important side effects.

The aim of this narrative review is to provide an overview of the possible use of TMS as a tool to investigate motor cortex excitability in sport. The search was carried out using the PubMed search engine, by entering the following keywords: Cortical excitability, Transcranial magnetic stimulation, motor cortex, TMS. We took into consideration studies carried out on athletes who played individual sports, which were members of team, regularly competing at national and international levels.

## 2. Transcranial Magnetic Stimulation (TMS)

**Figure 2:**
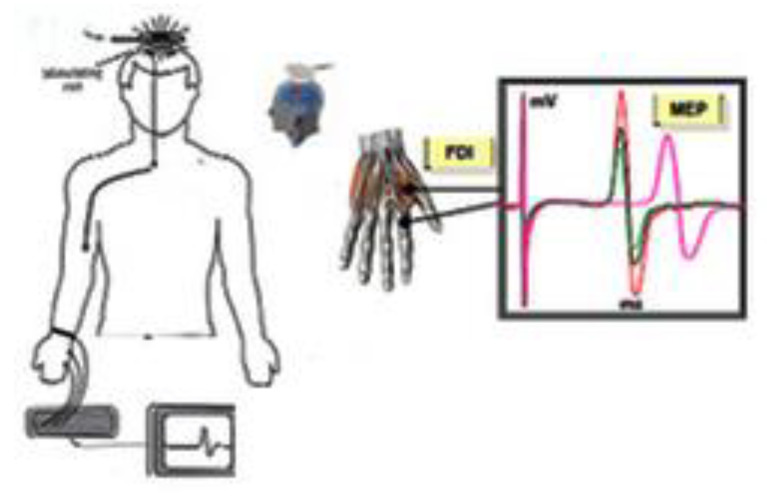
Motor Evoked Potential (MEP). Motor evoked potentials are the electrical signals recorded from the descending motor pathways or from muscles following stimulation of motor pathways within the brain.

**Figure 3:**
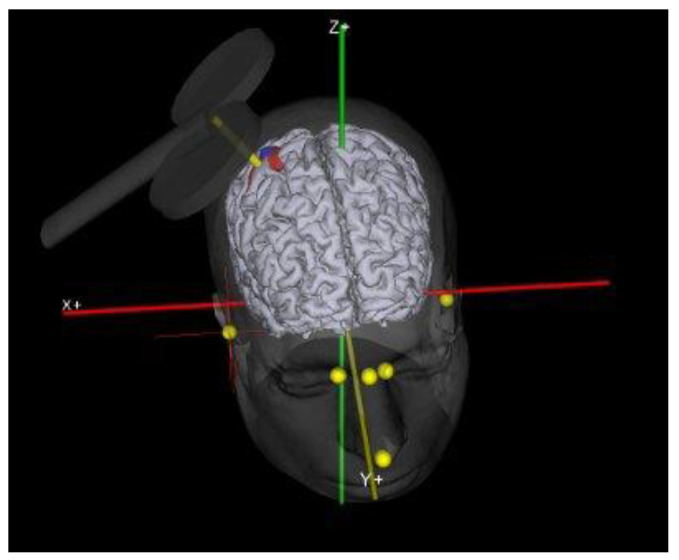
Coil Position. In this figure was show the exact coil location for motor cortex stimulation.

The TMS is a noninvasive method to investigate the CNS in the human [9]. Since its introductions close [13], TMS, has been used to study intracortical, cortico-cortical, and cortico-subcortical interactions [14]. In 1982, Polson, Barker and Freeston produced the first magnetic stimulator capable of stimulating peripheral nerves and in 1985 Barker, Jalinous and Freeston were the first to describe magnetic stimulation of the human motor cortex [13]. The information described above led to the development of the TMS. With this device, through a coil held on the scalp, magnetic fields are generated capable of inducing weak currents able to excite the underlying neural tissue. These currents cause activity in specific parts of the brain, with minimal discomfort, allowing us to study neural functions and interconnections in the intact human being. Brain stimulation techniques, as well as those of the peripheral nerve, trigger a series of events which, by depolarizing the neuron membranes, trigger the action potential. Experience from invasive stimulation during neurosurgery or epilepsy monitoring shows that stimulation parameters for the CNS are similar to those needed for peripheral nerve: short pulses with a duration of less than 1 ms and with an amplitude of few milliamperes. TMS methods for brain stimulation face the problem of delivering such a stimulus across the high resistance barrier of the periencephalic ‘layers’, including scalp, skull, meninges and cerebrospinal fluid [15]. The first brain stimulation studies were conducted using high voltage electrical stimuli through the use of electrodes placed directly on the scalp. This technique is known as transcranial electrical stimulation (TES) [16]. The TES did have the huge merit of introducing a neurophysiological technique for studying for the first time excitability and propagation properties along CNS fibers in intact and cooperative human beings [15]. However, the fields of application declined rapidly with the introduction of TMS because high-voltage TES is uncomfortable [13]. The ability of TMS to stimulate deep neural structures, such as the motor cortex, has enabled researchers to investigate the integrity of the brain to muscle pathway and the functionality of cortical networks [17]. To appreciate the potential of TMS, it is necessary to characterize the neuromuscular responses to cortical stimulation (Figure 2). The MEPs were elicited by positioning the coil tangentially to the scalp with the handle of the coil pointing backward and 45° laterally from the interhemispheric line (Figure 3).

Since neurons connecting to muscles in distinct regions of the body have their own geographical location across the motor cortex [18], it is possible to deliver magnetic stimuli to discrete collections of neurons relating to specific muscle groups. The TMS has been used also to study the human nervous system within clinical populations [19,20,21]; mechanisms of fatigue in small, isolated muscle groups [22,23,24,25]; corticospinal contributions during human gaitand acute neural adaptations following strength training [26,27,28,29]. In neurostimulation studies using TMS, magnetic pulses are delivered directly to the motor cortex and their MEPS is recorded on the muscle by surface electromyography. The intensity of magnetic stimuli is typically given as a multiple or a percentage of the resting motor threshold, which is the intensity to evoke members of a certain amplitude in a specified fraction of a series of consecutive demonstrations in a hand muscle. However, several studies show that the resting motor threshold in humans varies from subject to subject, so to obtain significant results it would be advisable to have an adequate number of subjects involved in the studies. The resting motor threshold (rMT) is the intensity of the stimulus necessary to evoke a muscular response; the rMT is used as target intensity for the following stimulations [30]. TMS induces electrical currents in the brain via Faraday’s principle of electromagnetic induction [31]. Faraday has shown that an electrical impulse that runs through a wire wound in a coil generates a magnetic field, and the speed variation of this magnetic field causes the induction of a secondary current in a nearby conductor. This is what happens with TMS, where an electrical stimulus, which reaches peak strength and decreases to zero in a short period of time (<1ms), is sent through the conductive wiring inside the TMS coil. The rapid fluctuation of this current produces a magnetic field perpendicular to the plane of the coil that similarly rises (up to about 2.5 T) and falls rapidly in time. This rapidly fluctuating magnetic field passes unimpeded through the subject’s scalp and skull and induces a current in the brain in the opposite direction of the original current [31,32,33,34,35]. When TMS is performed with the target muscle steadily contracting, it shows different results than when the muscle is relaxed. Muscle contraction has three main effects [36]: The threshold for evoking the motor response is reduced, the latency of the MEP is shortened, and the amplitude of the MEP is markedly increased [37]. A subthreshold stimulus followed by a suprathreshold test stimulus (S1) at interstimulus interval (ISI) of 1–6 ms, the MEP generated by the S1 is inhibited and this is known as short interval intracortical inhibition (SICI). On the other hand, the MEP generated by S1 is facilitated at ISI of 8–30 ms and this is termed intracortical facilitation (ICF) [38]. The underlying mechanisms for facilitation are not entirely understood but likely include increased cortical and spinal excitability [39]. With voluntary contraction, the resting potential of the anterior horn cell (AHC) is closer to a threshold, requiring less temporal summation of descending volleys, which means that the discharge can occur at an earlier I or D wave, thus shortening the onset latency. The increase of the compound muscle action potential amplitude indicates the recruitment of a greater number of spinal motoneurons. This could also be due to increased spinal excitability, increased synchronization of spinal motoneuron firing, or an increasing number of I waves bringing more AHCs to the threshold. In the past years, the deep brain stimulation (DBS) technique has been used to investigate movement disorders. DBS involves the implantation of electrodes in certain areas of the brain. These electrodes produce electrical pulses that regulate abnormal pulses. The amount of stimulation in deep brain stimulation is controlled by a pacemaker-like device placed under the skin in the upper chest. A wire that travels under the skin connects this device to electrodes in the brain. Neuroscience investigations have revealed that DBS may be correlated to several mechanisms including functional changes with neuronal activation or inhibition, neurotransmitter release, and long-term plastic changes in target and remote areas [40]. The DBS technique is more invasive than tms; moreover, DBS is mainly indicated for the treatment of some pathologies. Future studies exploiting the combined use of TMS and DBS in patients with movement disorders could lead to new treatment strategies for these patients.

## 3. Cortical Excitability and Physical Exercise

In recent decades, in order to understand how the brain networks build and optimize motor programs, responsible for the different types of muscle activity and related coordination [41], numerous studies have been performed that included the use of neuroimaging and TMS [42,43]. The ability of TMS to stimulate deep neural structures, such as the motor cortex, has enabled researchers to investigate the integrity of the brain to muscle pathway and the functionality of cortical networks [44]. Since MEP are readily measurable by electromyographic recordings on peripheral muscles, the investigation of cortical excitability has become the focus of numerous studies. The brain reorganization in human is highly dependent on the specific behavioral demands of the training experience.

### 3.1. Skill Training

As showed by Pearce et al., highly skilled racket players show larger hand motor representation and also showed increase in MEP amplitudes compared with less proficient players and nonplaying controls [45]. Moreover, Tyc et al. show that highly skilled volleyball players showed significantly larger and more overlapping representations of medial deltoid and carpi radialis muscles, compared to runners [46]. Furthermore, TMS could be suitable for investigating the effect of acute motor exercise on the excitability of the motor pathway [47]. In fact, the augmented amplitudes of MEP have been reported as a result of acute exercise bouts, substantiating the increased neuronal excitability during fatigue.

### 3.2. Fatigue

In sport competition, fatigue has a large influence on performance. The term fatigue refers to any exercise inducing loss of ability to exert force or power with a muscle or a muscle group [46,47,48,49]. This phenomenon seems to be due to changes in the excitability of the motor pathway both at central and peripheral levels [50,51,52,53,54]. During the execution of maximal voluntary contractions, fatigue results from both peripheral and central factors, which play an important role in the decline of strength which results from a sub optimal output from the primary motor cortex, which ultimately leads to sub-optimal firing rates of motor neurons. On the other hand, when an incremental exhaustive exercise is performed, a rapid decrease in muscle phosphocreatine and ATP occurs and consequent accumulation of metabolites such as pyruvate and lactate [55,56,57]. There are few reports on TMS and fatigue in sports-specific motor activities.

### 3.3. Aerobic and Anaerobic Exercise

The first study to show the possible use of TMS in sports and various kinds of everyday exercises was undertaken by Hollge et al. [58]. This authors investigated the changes in muscle response and in central motor conduction times after aerobic (climbing stairs and jogging), and anaerobic (press-ups, dumb-bell holding, and 400 m run) exercises. Exhausting strength exercises resulted in an important decrement in muscle response measured by electromyography with an relative improvement in cortical excitability, while no significant changes were elicited by aerobic exercises [59,60,61,62,63]. Other authors [64] investigated the fatigue-induced change in the corticospinal drive to back muscles in elite rowers compared to an untrained subject. These authors found an improvement in cortical excitability in elite athletes. Recently, in different investigations, were reported that, the excitability in the primary hand motor cortex investigated with TMS, is enhanced at the end of a maximal incremental test and that this improvement strongly correlates with the increase in the blood lactate concentration [65,66]. However, recently study shows that an increase of blood lactate is correlated to an enhancement of the cortical excitability evaluated with TMS. In fact, after fatiguing hand-grip exercise, there was an increase in blood lactate with a significant decrease in rMT and MEP amplitude in a trained subject (taekwondo athletes) and in an untrained subject (non-athletes). Compared to pre-exercise values, blood lactate strongly increased at the end of exercise in each group, decline after 3’ min, and recovered to the pre-exercise value within 10 min. However, as expected, in non-athletes’ blood lactate increase strongly compared to athletes. In this investigation was showed that a voluntary sub-maximal tonic contraction is associated with a significant increase in blood lactate level. This increase in blood lactate was a consequence of the relatively small muscle mass involved in the exercise coupled to the low-level work done during grip [66,67,68,69,70,71]. Regarding the relationship between excitability and blood lactate, it has been suggested that when lactate increases due to strenuous exercise, the brain absorbs a similar amount to that of glucose. In this investigation, the reduction of rMT is maximal at the end of maximal exercise in parallel with the increase of blood lactate. Furthermore, also at the end of maximal exercise, and in parallel however non-athletes show higher depression of MEP amplitude compared to athletes at the end of exercise (−22.97% vs. −71.15%). Furthermore, in non-athletes, significant decrease emerged after 3 min of the end of exercise, while in athletes this differences disappeared. Therefore, it seems that, besides a possible role of exercise-elicited reduction of the blood flow in the cortex, the exercise-induced increase of blood lactate could be capable, in the frontal lobe, of worsening the performance in the prefrontal cortex and improving the excitability of motor cortex [72,73].

## 4. The Use of TMS in Sport Science

The use of the TMS for research purposes in the motor and sports field is of great interest as it is applied to investigate post-exercise facilitation, central fatigue, sensorimotor integration, motor coordination, and neuronal plasticity. For example, with TMS, it was possible to demonstrate that when a subject performs a voluntary non-maximal muscle contraction, the corticospinal path to the muscle is facilitated [74]. Additionally, other neurostimulation studies with TMS have shown greater improvement in MEP for precision movement than for general gripping tasks, and this seems to be due to greater recruitment of pyramidal neurons [75]. Instead, there are conflicting arguments regarding the facilitating effects during a voluntary contraction of the ipsilateral orneighboring homonyms muscles [43]. However, in addition to the acute effects of motor activity, long-term effects of MEP enhancement can also be appreciated. In fact, Brasi-Neto et al. show that 10-s activation could lead to post-exercise facilitation, which decayed to the baseline over 2 to 4 min [76]. Since these effects were not present after the magnetic stimulations, the researchers hypothesized that these are the changes in the intracorticular plastics. Hollge et al. (1997) were the first to apply TMS to the study of dynamic exercise [58]. Those authors found significant decreases in MEP amplitude evoked in the primary muscles associated with exhaustive 400 m running, press-ups and dumbbell holding. This decrease were described as a central failure because responses to peripheral nerve stimulation were unchanged [58]. Confirming this CNS impairment, reduced intracortical facilitation was found after pull-ups to task failure, reflecting a decreased excitability of interneuronal circuits within the motor cortex [58]. Others authors shoed reduced MEP amplitudes of both the quadriceps and diaphragm after maximal incremental treadmill exercise, with no change in the response to peripheral nerve stimulation [77]. Transcranial magnetic stimulation has also been used to assess supraspinal fatigue of small muscle groups working in isolation. Goodall et al. (2012) used TMS to evaluate supraspinal fatigue of the knee-extensor muscles in response to sustained, high-intensity cycling in normoxia and acute severe hypoxia. Cortical voluntary activation declined after exercise in both conditions, but the decline was two-fold greater in hypoxia. Recently, Moscatelli et al. (2016), investigated the relationship between blood lactate and cortical excitability in taekwondo athletes. In this study, the authors show that blood lactate seems to have a greater influence in athletes compared to untrained subjects. It seems that, during extremely intensive exercise in athletes, lactate may the onset of fatigue not only by maintaining the excitability of muscle but also by increasing the primary motor cortex excitability more than in non-athletes [41]. Collectively, these findings suggest that TMS has the potential to quantify the contribution of central processes to fatigue of limb locomotor muscles. A recent investigation showed that, after 8 weeks of aerobic training, there was a significant increase of distance covered during Cooper’s test and a significant increase of VO2max; there was also an improvement in resting motor threshold, MEP latency and ME amplitude improvement [78]. Transcranial magnetic stimulation can be used to investigate physiological states other than fatigue. For example, it is well established that neuromuscular adaptation readily occurs as a result of resistance exercise training [17]. The M1 is heavily involved in voluntary contraction of skeletal muscle and shows a high degree of plasticity, or capacity to change quickly, with motor practice [42,43,44]. In a classic example, Muellbacher et al. (2002) showed that 20 min practice of a ballistic pinching task elicited a significant improvement in task performance [79]. The improvement in task performance was accompanied by an immediate increase in the corticospinal response, demonstrating that M1 has an adaptive role in the consolidation of motor tasks (Table 1).

Therefore, TMS enables a greater understanding of the behavior of the corticospinal tract in ‘top-down’ paradigms, where the effect of motor skills on corticospinal plasticity and neuromuscular adaptation can be examined. The remainder of this section will explore some potential applications of TMS for the investigation of M1 plasticity during and following different experimental paradigms, including task-specific contractions and resistance exercise training.

An interesting study was recently published that shows the effects of tDCS using the Halo Sport device on repeated sprint cycling ability and cognitive performance. The authors found that by using this device, the power delivered by repeated sprint cycles was improved. Interest in the possible ergogenic effect of noninvasive brain stimulation is growing and therefore in the future it could be useful to conduct new experiments to evaluate the impact on learning and motor performance [80,81,82].

## 5. Conclusions

The introduction of neurostimulation techniques has had a positive impact on scientific research in various fields. Since these techniques are not invasive, and at the same time they are safe, numerous studies can be carried out in different population groups. Regarding the motor and sports field, it is likely that in the future more and more studies involving the use of neurostimulations will be carried out to understand the effects of training in the short and long term.

## Figures and Tables

**Table 1:** TMS in physical exercise. In this table are sreported the research performed to investigated the relationship between cortical excitability and physical exercise.

Authors	Type of Sport	Type of Exercise	Main Findings
Jensen et al., 2005 [28]	Original research	Strength training	The results of this investigation show that increased corticospinal excitability may develop over several weeks of skill training and indicate that these changes may be of importance for task acquisition.
Moscatelli et al., 2016 [41]	Original research	Karate	Karate athletes show higher corticospinal excitability compared to non athletes indicating the presence of an activity-dependent alteration in the balance and interactions between inhibitory and facilitatory circuits determining the final output from the M1
Moscatelli et al., 2016 [42]	Original research	Karate	The practice of competitive sports affects central/peripheral nervous system. Subjects that showed higher cortical excitability showed also higher velocity at which the neural signal is propagated from the motor cortex to the muscle and consequently better reaction time.
Moscatelli et al., 2016 [44]	Original research	Taekwondo	The results of this study show that blood lactate seems to have the greater effect in taekwondo athletes compared to untrained subjects. During extremely intensive exercise in taekwondo athletes, lactate may delay the onset of fatigue not only by maintaining the excitability of muscle, but also by increasing the excitability of the primary motor cortex more than in non-athletes.
Tergau et al., 2000 [47]	Original research	Lifting	Double-pulse TMS gives access to the motor cortex independently of spinal or peripheral mechanisms, reduced Intra Cortical Facilitation reflects decreased excitability of interneuronal circuits within the motor cortex.
Coco et al., 2014 [52]	Original research	Intensive isometric exercises	The relation between blood lactate and the amplitudes of motor-evoked potentials showed a significant direct proportionality.
Höllgeet al., 1997 [58]	Original research	Aerobic and anaerobic exercise	This investigation show the possible use of TMS in sports medicine, indicating that only exhaustive or strength exercises result in reduced MEPs.
Ljubisavljević et al., 1996 [59]	Original research	submaximal isometric voluntary contraction	The increase in MEP magnitude after the sustained 60% maximal voluntary contraction may indicate residual changes in cortical activity after fatiguing contraction.
MaKay et al., 1996 [60]	Original research	Isometric maximal contraction	These results of this investigation suggest that MEP and SP might have common sources of facilitation during maximal voluntary contraction and that inhibitory mechanisms remain focally augmented following a fatiguing maximal voluntary contraction.
Fulton et al., 2002 [64]	Original research	Rowers	There were no differences in MEP depression or latency between elite rowers and non-rowers after intense exercise. The authors conclude that the smaller degree of MEP depression in the elite rowers after light exercise reflects less central fatigue within corticospinal control pathways than that seen in the non-rowers.
Coco et al., 2010 [70]	Original research	Cycling	In this study was observed that an increase of blood lactate is associated with a decrease of motor threshold, that is, an enhancement of motor cortex excitability. The authors conclude by hypothesizing that in the motor cortex the lactate could have a protective role against fatigue.
Moscatelli et al., 2020 [78]	Original resea	Aerobic exercise	This study shows that aerobic activity seems to induce changes in cortical excitability if performed for a period longer than 4 weeks, in addition to typical cardiorespiratory benefits in previously untrained males
Percivalle et al., 2010 [68]	Original research	Maximal exhausting exercise	The authors observed a similar enhancement of excitability of primary motor cortex, concomitantly with an increase of blood lactate, in both young male and female athletes. However, the improvement was significantly higher in women than in men, suggesting a greater sensitiveness of female cerebral cortex to blood lactate.
Cros et al., 2007 [75]	Original research	Isometric contraction	The timing of central conduction was different depending on functional role of the target muscle, as either agonist or joint fixator. These results indicate that the architecture of motor plans remain grossly undisrupted by cortical stimulation applied during voluntary motor behavior.
Brasil-Neto et al., 1994 [76]	Original research	Isometric and isotonic exercise	The results are similar to those found at the neuromuscular junction in myasthenia gravis and are consistent with a reduced safety factor of cortical synaptic transmission in central nervous system fatigue.
Verin et al., 1985 [77]	Original research	Incremental treadmill exercise	The results of this study confirm significant depression of both diaphragm and quadriceps MEPs after incremental treadmill exercise.
